# Supplementary material for: Oxidative stress response in children undergoing cardiac surgery: Utility of the clearance of isoprostanes
Source: PLoS One. 2021 Jul 6;16(7):e0250124. doi: 10.1371/journal.pone.0250124 (PMC8259993; doi:10.1371/journal.pone.0250124)
Supplement: S1 Data — (DOCX) [file pone.0250124.s001.docx]

S1 Data

Title: Surgical Procedures

| Surgical repair | Number (%) |
| --- | --- |
| Aortic arch repair | 2 |
| Arterial switch operation | 8 |
| ASD repair, primary closure | 1 |
| Blalock Taussig shunt | 9 |
| Coarctation repair | 13 |
| DORV, intraventricular tunnel repair | 1 |
| Glenn procedure | 1 |
| Mustard procedure | 1 |
| Norwood Procedure | 1 |
| PA banding | 3 |
| Ross Konno procedure | 1 |
| TAPVR repair | 2 |
| TOF repair | 6 |
| Truncus arteriosus repair | 2 |
| Valve replacement, mitral | 1 |
| VSD repair, patch | 8 |
| Others | 3 |

ASD: Atrial Septal Defect; DORV: Double Outlet Right Ventricle; PA: Pulmonary banding; TAPVR: Total Anomalous Pulmonary Venous Return; TOF: Tetralogy of Fallot; VSD: Ventricular Septal Defect.
