# Supplementary material for: Oxidative stress response in children undergoing cardiac surgery: Utility of the clearance of isoprostanes
Source: PLoS One. 2021 Jul 6;16(7):e0250124. doi: 10.1371/journal.pone.0250124 (PMC8259993; doi:10.1371/journal.pone.0250124)
Supplement: Attachment — Submitted filename: RESPONSE TO REVIEWERS.docx [file pone.0250124.s003.docx]

**Reviewer #1:** This is an interesting study that reports that infants undergoing cardiac surgery have oxidative stress as measured by increases levels of 8-iso-PGF2a in their urine before, during, an in the few days after surgery. When levels of this marker were compared to various clinical parameters and outcomes, they found that higher oxidative stress was correlated with use of cardiac bypass (CPB) and was higher in patients with d-TGA, who they say had extreme cyanosis before surgery. High levels of this marker are associated with longer ventilation and ICU days, increased inotropic support, and habituation score. They conclude that infants undergoing cardiac surgery have increased oxidative stress that may play a role in post-operative morbidity. However, there are many pieces of data missing in this report that make this report incomplete and difficult to interpret. This lessens enthusiasm for its publication in the current form.

Major issues:
1. The authors need to make a better argument that infants have poor “cardiovascular bypass tolerance.” It is unclear what they mean by this and the references they give seem only to discuss this in relation to oxidative stress, which, as they state, has uncertain relation to clinical outcomes. In fact, some data suggests that neonates may have better tolerance to cardiac bypass.

*The reviewer is right, pediatric patients (including neonates) may have a good tolerance to cardiac bypass. What we were trying to state was that Cardiopulmonary Bypass (CPB) could increase the oxidative stress response, as we have proved in our paper. The sentence that was written in the paper could lead to a misunderstanding, so we have rephrased to make it clearer. (lines 117-119)*

2. There are two groups presented, neonates and pediatric patients, and some neonates but all pediatric patients underwent CPB. Some analyses compare data based on age (neonate vrs pediatric) while other analyses are for CPB vrs no CPB. The analyses should consistently include both comparisons. For example, the authors state that the immature state of anti-oxidant defenses is revealed in their data and this is detrimental to infants undergoing surgery, but they state in the text (page 12, lines 215-218) say that this is not the case, while the data in Figure 2 suggests that CPB is a larger contributor to elevated oxidative stress. However, since all of the pediatric patients while only some neonates underwent CPB, it might be that the interaction of age and CPB would be revealing. In addition, might the data on d-TGA patients be significant because they underwent CPB?

Thank you for your comment. As we have explained with some of the included references ( Torres-Cuevas et al, 2017), the antioxidant system is very immature during the first month of life. Consequently, the antioxidant response is decreased, and the oxidative damage seems to be more important during this period, achieving its maturity progressively.

Moreover, CPB surgery seems to be one of the most important factors contributing to oxidative stress damage. Despite the fact that patient age seems to be a protective factor, the older the patient at time of surgery, the lower the oxidative stress response. To improve the understanding of these results, and following the suggestions of the reviewers, we have included a better explanation of this and a complementary figure (Figure 2b) to make it more understandable. Included in line 340 to 351.

*“A regression analysis was performed to correct for age as a possible confounder, which showed no differences in the levels of 8-iso-PGF2α (p=0.371). Amongst all CPB patients, neonates had overall increased OS response both pre- and post-surgery compare to pediatric patients with differences reaching statistical significance at 24 hours post-surgery (p=0.0421) (Fig 2B)”.*

New Figure 2:

Another interesting point that the reviewer suggested is the effect of CPB on d-TGA patients since all of them required CPB surgery for correction. A multivariable regression to exclude CPB effects as a confounder has been performed, and the differences between groups persists (p=0.0214). To clarify that, we have also included the analysis of OS considering cyanosis classification only in patients that require CPB.

This new analysis has been included in lines 366-367.

*“A regression analysis to exclude CPB effects in TGA patients as a confounder was performed, and the differences between groups persisted (p=0.0214). “*

3. More information should be given about the patient characteristics and state before surgery, such as comorbid conditions, clinical condition, other procedures, and the defects themselves with their surgical approach and need for bypass or not. Only limited data on CHD lesions is presented in Tables 1 and 2, but Table 2 should break down diagnoses by age category and which diagnoses did not receive cardiac bypass as the low amount of bypass in neonates seems odd. For example, they make much of the fact that patients with d-TGA have profound cyanosis that makes them more likely to have oxidative stress. However, such cyanosis is usually corrected with an atrial septostomy and treatment with prostaglandins, increasing systemic saturation and stabilizing the patient. Such patients are not usually rushed to the operating room when unstable. Furthermore, other congenital heart defects can present with cyanosis as profound as seen in d-TGA. Therefore, their explanation of why d-TGA patients had higher levels of 8-iso-PGF2a in the discussion (page 15) is speculative and not probably logical.

*Considering reviewer suggestions, some pre-surgical patient characteristics have been included in table 1. Moreover, table 2 has been rebuilt showing diagnosis by age category and kind of surgery.*

*“Pre-surgical levels of OS among the three cyanosis groups (non-cyanotic, mild-cyanotic and TGA) were not statistically different (p=0.359), including in post-hoc analysis, confirming that the basal levels of OS biomarkers were similar among these groups before surgery. The differences appeared in the post-operative period. Those patients with a severe degree of cyanosis caused by Transposition of the Great Arteries had the highest post-operative 8-iso-PGF2α levels (p=0.017). Moreover, in the post-hoc analysis of post-surgery OS levels, TGA patients’ OS levels were higher when compared to non-cyanotic patients (p=0.019) and there was a tendency when compared to mildly-cyanotic patients (p=0.095). No differences were found in OS levels at 24 hours (p=0.9218) (Fig 3)”.*

*“All these data suggest that during surgery, those changes in oxygenation, perfusion, hemodilution, and other variables related to oxidative status had a greater effect in those patients with a pre-surgical cyanotic condition, especially in those with TGA.”*

*To clarify this, we have included a more accurate statistical description of the results in lines 356-366, and we have also included additional explanations in the discussion lines 572-575.*

4. Levels of 8-iso-PGF2a are reported as ng/mg creatinine. However, at least creatinine if not other measures of renal function should be reported. Based on the data reported, one questions whether the clearance of 8-iso-PGF2a is dependent on renal function, and that all results reported in this manuscript could be due to low cardiac output yielding poor renal function and thus slow clearance of 8-iso-PGF2a. However, I do recognize that such as state might also prolong systemic oxidative stress for the reasons given by the authors.

*We report levels of 8-iso-PGF2a in “ng/mg creatinine” following the instructions of the ELISA kit. All levels of proteins in urine must be normalized by another protein, to prevent dilutional influences. 8-iso-PGF2 levels were usually normalized with urinary levels of creatinine. With this normalization the effect of ultrafiltration or possible renal function alterations were avoided.*

5. Although cSO2 is reported, arterial saturation and p02 and measures of cardiac output are not reported. Not only might cSO2 depend on these parameters, but these values might also reflect systemic oxygen delivery, which could affect oxidative stress throughout the body. This would also clear up the descriptions of “typical cyanosis” and “extreme cyanosis” that are used in the text.

*We completely agree with the reviewer’s comments. Unfortunately, data on preoperative oxygen saturation and continues hemodynamics are not available since the study data was collected before the upgrade of the unit monitoring system as well as the implementation of the new electronic medical report platform. Both systems would have helped to better understand the underlying CHD and the preoperative oxygenation state.*

*Cyanosis in congenital heart disease is due to the existence of either intra- or extra-cardiac shunts resulting in deoxygenated blood entering the systemic circulation and thus, generating different levels of cyanosis. Cyanotic congenital heart disease (CCHD) has been previously classified as follows: 1) CCHD with decreased blood flow, typically classified as tetralogy of Fallot physiology (typical cyanosis) and; 2) Cyanotic congenital heart disease with increased blood flow, typically classified as transposition physiology (extreme cyanosis).*

*We have included in the updated version of the manuscript an explanation of this classification published by Rohit & Shrivastava in 2018, to clarify this topic (lines 146-151)*

6. Very little is presented on the EEG data, and no correlation is made to patient characteristics, including age and use of CPB, which may be important relations.

*According to the reviewer suggestion some more data about aEEG have been included in the text. aEEG patterns and the presence of electrical seizures has been analyzed in relation to CPB and age. This explanation appears in lines 273-278.*

7. The figure legends need to be expanded to actually explain the Figures, including mentioning statistical and graphical parameters (N, +/- SD or SEM, statistical test used). Furthermore, only Figure 3 has markers of statistical significance (but with the N of each group not presented), and it is reported in the text that the post-surgery 8-iso-PGF2a levels are significantly different in Figure 1a, but given the data presented it is hard to believe this without more information.

*Figure and figure legends have been modified. Statistical significance has been included and some more explanations too.*

Minor issues:
1. There are some spelling and grammatical errors. The manuscript should be gone over to eliminate these. Some examples include:
a. “response,” “isoprostanes,” and “clearance” are mis-spelled in the title.
b. Page 2, line 40, “measure” should be “measured”
c. Page 3, line 63, “increase” should be “increased” or “increase in”
d. Page 4, Line 91, “Material ad methods” should be “Materials and methods”
e. Page 6, line 120, “Continues” should be “Continuous”
f. Page 10, line 199, “were exposed” should be something like “are evident”
g. There are other examples

*Thanks for the corrections, all of them have been changed in the text and we have also reviewed the manuscript carefully again to correct other errors.*

2. The statistics section of the materials and methods states that data were checked for normality, but the only multiple comparison test mentioned was Kruskal-Wallace. Was all data non-parametric?

*Yes, most of the analyzed variables were non-parametric. Despite that, previous to the analysis their normality was checked, and we included it in the statistics description.*

3. In the text, page 10, lines 197-198, the lower cSO2 values are reported to be 68% while the higher are 60%; please correct this error.

*Thanks for the suggestion, we have changed it in the text.*

4. The authors should explain what they mean by the statement “These results are not surprising given the smaller size of the patients in relation to the bypass circuit. . .” (Page 15, lines 286-287).

*This statement is a little bit confusing, so following the reviewer’s suggestion we have changed it.*

Reviewer #2: This manuscript examines the role of oxidative stress (OS) in the post-op course of neonates and infants with congenital cardiac defects. The topic is an important one as oxidative stress may be a critical determinant of post-operative outcomes and may be a therapeutic target in the strategy to improve outcomes. The choice of 8-isoprostaglandine-F2α as a readout of oxidative stress exposure is reasonable. The cohort is relatively small in size and diverse, complicating interpretation of the findings. Comparison of the two subgroups, the neonates and the infants, is heavily confounded by different clinical factors and operative approaches. For instance, only a third of the neonates underwent CPB which will have an important effect on inflammatory activation, endothelial injury and oxidative stress. The description of profound cyanosis and acidosis in the dTGA patients is concerning regarding how those patients were managed. Commonly, balloon atrial septostomy is performed to prevent severe cyanosis and acidosis and a period of stabilization is allowed before surgery. In addition, the surgical complexity is relatively low with most patients being STAT category 1 for surgical complexity which will affect oxidative stress burden.

Major concerns:
1. The neonatal and pediatric patient populations are different enough that any statements regarding reflection effect of age on oxidative stress management and capacity in this study must be made with caution.

*The reviewer is right, and this is one of the hot topics of our paper. The effect of maturation over the oxidative stress response is of paramount importance, especially in the youngest patients. In our population, neonatal patients (those less than one month old) show significant differences in the clearance of Isoprostanes. This may reflect the immaturity of this system during the first days of life, as it also has been reported in other pathologies*

*Another point that is also interesting regarding age is that no statistical differences were detected in OS levels prior to surgery or immediately after surgery. Considering these data, we could hypothesize that the basal and post-surgery responses are similar in both age groups, and that the major differences appear when the antioxidant systems have to be used, as these systems are more efficient in the older patients.*

*A final conclusion about this point has been included in the paper line 647-653.*

2. “Typical” and Extreme” cyanosis is not well defined and the management of dTGA patients with regard to frequency of and indications for BAS are not described.

*Considering reviewer suggestion we have included a better explanation of the classification .*

*Cyanosis in congenital heart disease is due to the existence of either intra- or extra-cardiac shunts resulting in deoxygenated blood entering the systemic circulation and thus, generating different levels of cyanosis. Cyanotic congenital heart disease (CCHD) has been previously classified as follows: 1) CCHD with decreased blood flow, typically classified as tetralogy of Fallot physiology (typical cyanosis) and; 2) Cyanotic congenital heart disease with increased blood flow, typically classified as transposition physiology (extreme cyanosis).*

*We have included in the updated version of the manuscript an explanation of this classification published by Rohit & Shrivastava in 2018, to clarify this topic (lines 146-151)*

*Frequency and indication of BAS have been added in Material and Methods as well as in table 1.*

3. The number of patients requiring deep hypothermic circulatory arrest is not described.

*We have included these data in Material and Methods line 200-201.*

4. Particularly for the neonatal cohort, it would be important to know if there were any that were born prematurely as this may affect both OS response and clinical outcomes.

*Thanks for the suggestion, these data have been included in table 1.*

5. To more directly compare the neonatal and pediatric cohorts, a comparison of OS levels and clearance should be performed specifically in those neonatal subjects who underwent CPB.

*Considering the reviewer suggestion, this analysis has been performed and included in the text lines 348-351 and in Figure 2B.*

6. In the subdomain analyses, 8-isoprostaglandine-F2α descent is likely confounded with CPB exposure in the neonates and it is unclear from the data presented if the effects are due to OS or other effects of CPB.

*All pediatric patients in our population require CPB surgery. When clearance of OS was analyzed only in neonatal patients to consider the effect of 8-isoprostaglandine-F2α descent, no differences were found (p=0.3392)*

*Thanks for the suggestion, we have clarified this point in the text, lines 500-503.*

Minor comments:
1. In abstract, the “(8.04 ng/mg Cr were higher than those pre-operatives 5.7 [6.4-10.3] …” is missing a ).

*Thanks, this has been changed in the text.*

2. Typo in Methods and Materials

*Thanks, this has been corrected in the updated version of the manuscript.*

3. The clearance formula is not properly displayed.

*Thanks, this has been modified in the updated version of the manuscript.*

4. In line 197, the “lower cSO2 values…” line does not make sense as written.

Thank you for pointing at this. We have identified the grammatical mistake and we have rephrased the sentence in the updated version of the manuscript.
