# Supplementary material for: Oxidative stress response in children undergoing cardiac surgery: Utility of the clearance of isoprostanes
Source: PLoS One. 2021 Jul 6;16(7):e0250124. doi: 10.1371/journal.pone.0250124 (PMC8259993; doi:10.1371/journal.pone.0250124)
Supplement: Attachment — Submitted filename: Response to the reviewers_FINAL.docx [file pone.0250124.s004.docx]

Additional Editor Comments (if provided):

Please, address the concerns described by Reviewer 1. All of them should be answered properly.
Secondly, send to a native english speaker and/or provide a review of the manuscript to avoid english errors (e.g., proof reading services). Provide also a proof of english correction.

Thank you very much for the great job you have done until now.

*Dear (name of the editor), thank you for the thoughtful revision and for all the comments and suggestions you have made. We have addressed all the reviewers’ questions and we have prepared a final manuscript version that we are attaching along with a detailed point by point response to the reviewers’ comments. Our colleague, Stephanie Hadley, MD, current pediatric resident at Boston Children’s Hospital and former pre-doc intern in our research group at Children’s Hospital of Barcelona, has undergone a thoughtful revision of the manuscript in order to avoid English errors.*


Reviewers' comments:

Reviewer's Responses to Questions

6. Review Comments to the Author

Please use the space provided to explain your answers to the questions above. You may also include additional comments for the author, including concerns about dual publication, research ethics, or publication ethics. (Please upload your review as an attachment if it exceeds 20,000 characters)

Reviewer #1: This remains an interesting study that reports that infants undergoing cardiac surgery have oxidative stress as measured by increases levels of 8-iso-PGF2a in their urine before, during, an in the few days after surgery. When levels of this marker were compared to various clinical parameters and outcomes, they found that higher oxidative stress was correlated with use of cardiopulmonary bypass (CPB) in neonates immediately after surgery and was higher in patients with d-TGA. High levels of this marker are associated with longer ventilation and ICU days, increased inotropic support, and habituation score. They conclude that infants undergoing cardiac surgery have increased oxidative stress that may play a role in post-operative morbidity and that this is age dependent—older infants (>1 month of age) are less likely to have elevated oxidative stress as evaluated by urine 8-iso-PGF2. Although the authors have been responsive to the two reviewers, there remain problems with this work that need to be addressed. Please also note that some of these points were commented on by both reviewers independently.

1. The authors should reconsider their use of pediatric, infant, and neonate to describe patient groups. Pediatric is a more general term, whereas the authors are really evaluating a difference between neonates < 1 month of age and older infants.

*Thanks for the suggestion, the reviewer is right “pediatric” perhaps is not the best term to define the older group that includes those patients older than one month. We have renamed this group as the “infant” group.*

*This has been changed throughout the text.*

2. Explanations of some of the comparisons remains remain unclear. For example, the statistics reported in lines 242-248 and in Figure 3 are confusing. What is the test used to compare post-operative levels of TGA patients to get a p=0.017 and to what are they compared? It is then stated that in "post-hoc" analysis, TGA patients are different from non-cyanotic (p=0.019) and mildly cyanotic (p=0.095) patients. How is this different from the previous sentence?

*Thanks for the comment, as the reviewer noted, there was a mistake in how the sentence was written. The correct report of the statistics should be:*

*“In the post-operative period, however, there were significant differences between groups (p=0.017); in post-hoc analysis of post-surgery OS levels, TGA patients’ OS levels were higher compared to non-cyanotic patients (p=0.019) and trended higher than typically-cyanotic patients (p=0.095).”*

*The statistical test used for the analysis was Kruskal-Wallis test with a post-hoc analysis.*

3. Cyanosis: Note that both reviewers questioned the authors’ use of cyanosis levels, and I continue to find the distinction of levels of cyanosis not appropriate. In the first paragraph of the Discussion, which is a nice summary of the results, the clinical entity of TGA is said to be different from other diagnoses, but throughout the text, the authors continue to use the terms mild/typical and severe/extreme cyanosis. Although they provide a review reference for this (which I could not get), their use of the terms (d-TGA is extreme cyanosis while all other cyanotic lesions are not) is not standard and will be confusing to the general reader and discounted by most pediatric cardiologists. Many patients with d-TGA have less cyanosis that those with the other lesions listed in Table 2, and some of them (HLHS, TAPVR) can have cyanosis AND increased pulmonary blood flow, like in d-TGA. If the authors are going to define levels of cyanosis, then they should report % hemoglobin saturations or partial pressures of oxygen. The explanation that these data are not available due to the lack of an electronic medical record is nonsense. Pre- and intra-operative saturations and blood gasses should be present in the paper medical record, like other reported data (e.g., intra-operative temperature). The fact that those with d-TGA have higher levels of OS in the immediate post-operative period is interesting and should be reported, but is it also interesting that OS in patients with d-TGA is not significantly different from other neonates with “typical cyanosis,” making one wonder if perhaps there is a correlation between actual cyanosis (saturation, pO2) and post-op OS. Therefore, I believe you should get these data, as the level of pre-operative (and post-operative for that matter) cyanosis could play a role in OS, as the authors point out in the discussion about oxygenation during reperfusion after surgery.

*Following the reviewer’s suggestion, we have identified pre- and post-surgical hemoglobin saturations and partial pressures of oxygen in the medical records of our patients.*

*We have incorporated these values to include an analysis of these parameters together with our classification of cyanosis.*

*“To further support these cyanosis classifications, pre-surgical and post-surgical percentages of hemoglobin saturation and partial pressures of oxygen were analyzed. Patients with no cyanosis had a mean percent hemoglobin saturation of 98.6% (+/-1.4), those with mild cyanosis had a mean of 91.5 % (+/-7.38) and patients with TGA 79.6% (+/-7.9) (p= 0.001). Pre-surgical paO2 levels were also analyzed. Patients with no cyanosis had a mean of 145 mmHg (+/-65), patients with mild cyanosis had a mean of 82 mmHg (+/- 49) and patients with TGA 48.3 mmHg (+/-9.2) (p= 0.001), as demonstrated in figure 1a. Post-surgical percentages of hemoglobin saturation and partial pressures of oxygen were not statistically different amongst groups (p= 0.1041 and 0.2399, respectfully), as seen in figure 1b.”*

*We also have included a new figure:*

*These data support our hypothesis that reoxygenation plays an important role in the generation of oxidative stress products.*

*We have included this new analysis in the text to clarify this point.*

4. Table 1 needs to be modified.

a. The cell for STAT category needs to be justified to the left.

*We have included this correction in the new version of the manuscript.*

b. There should be more explanation somewhere about the surgeries performed (perhaps as a supplement). Although it is now nice that table 2 describes the diagnoses, based on these diagnoses, I would expect that more patients would have undergone cardiac bypass, unless there is a higher percentage than expected based on current care of patients undergoing palliative placement of systemic to PA shunts. Therefore, more information than a category of “Other” should be provided.

*Thanks for the suggestion, this information has been included as supplemental material.*

*Supplement 2*

c. Please explain why so many neonates were treated preoperatively with sub atmospheric oxygen.

*Preoperative measures aiming to achieve a balance between pulmonary flow and systemic flow is of paramount importance in the preoperative stabilization of many patients during the first days of life. Nitrogen* [*inhalation therapy*](https://www-sciencedirect-com.sire.ub.edu/topics/medicine-and-dentistry/inhalational-drug-administration) *reduces the fraction of inhaled oxygen, inducing hypoxic vascular* [*vasoconstriction*](https://www-sciencedirect-com.sire.ub.edu/topics/medicine-and-dentistry/vasoconstriction) *and PVR, and thus preventing pulmonary over-circulation while improving cardiac output and cerebral blood supply.*

*In this study, 16 patients received this therapy; nine patients with aortic coarctation and interventricular septal defect, 2 patients with Truncus arteriosus, 1 patient with DORV, 2 patients with HLHS (1 Norwood and 1 Hybrid correction), 1 with tricuspid atresia and 1 with pulmonary atresia.*

5. The definition of abnormal intra-operative EEG patterns needs to be stated, since there were no differences in seizures.

*Following the reviewer’s suggestion, we included a better explanation of abnormal intra-operative EEG patterns.*

*“Background patterns were analyzed in accordance to Hellström-Westas classifications. They were classified as continuous normal voltage (CNV), dis- continuous normal voltage (DNV), burst suppression (BS) and continuous low voltage. CNV and DNV were both considered normal, as even healthy infants may show discontinuous activity during sleep. Electrographic seizures were also identified.”*

*This part has been included from line 169 to 173.*

6. In lines 207-208, should the sentence start: "The median cSO2 AFTER surgery. . ."

*Thanks for pointing out this mistake. Please see the correct sentence in the new version of the manuscript.*

7. It appears the the CBP and no CBP headings in Table 3 are misplaced, since the text states that the mean sats for CBP and no CBP were 68 versus 60% while the numbers in the table reflect the opposite condition.

*The headings are not misplaced. The numbers that appear in table 3 represent the total amount of minutes (mean) that patients stayed in each condition. The mean saturation refers to cerebral region oxygen saturation during the intervention.*

8. The authors have disregarded my comment about renal function and clearance. This should be addressed. 8-iso-PGF2a levels will depend on production and renal clearance (presumably there are no other methods of clearance), so if renal function is decreased, then this could explain elevated levels. It is not unusual that infants suffered acute kidney injury after cardiac surgery, and the effects of this may not be present for a few days because monitoring markers other than BUN and creatinine of this are only being investigated now. Therefore, the authors should report if the patients suffered clinical AKI and the creatinine levels independent of 8-iso-PGF2a levels.

*Sorry we failed to address this important issue in our previous revision.*

*All 8-Iso-PGF2 levels were normalized using urine-creatine levels in order to avoid renal failure as a possible confounder. Moreover, in order to better address this issue, we have performed an independent correlation analysis of 8-iso-PGF2 levels and creatinine levels. Only one of the patients included required renal replacement therapy during the post-operative period.*

*We include the following part in the manuscript lines 246-251.*

*“Renal function was also analyzed as a possible confounder. There was no correlation between presurgical levels of creatinine and presurgical levels of 8-iso-PGF2 (p=0.3925). Similarly, no correlation was found between post-surgical levels of creatinine and immediate post-surgical levels of 8-iso-PGF2α (p= 0.8341), nor with levels of 8-iso-PGF2α at 24 hours post-surgery (p=0.2251).”*

Minor issues:
1. There are still some spelling and grammatical errors.

Reviewer #2: There remain some inherit limitations based on the cohort but the authors have done a nice job of addressing the concerns raised in the previous review. It will be hard to know whether slower clearance really is slower clearance or ongoing production.
